# Supplementary figures and images for: Establishment and Expression of Cytokines in a Theileria annulata-Infected Bovine B Cell Line
Source: Genes (Basel). 2019 Apr 30;10(5):329. doi: 10.3390/genes10050329 (PMC6562936; doi:10.3390/genes10050329)

## Slide 1
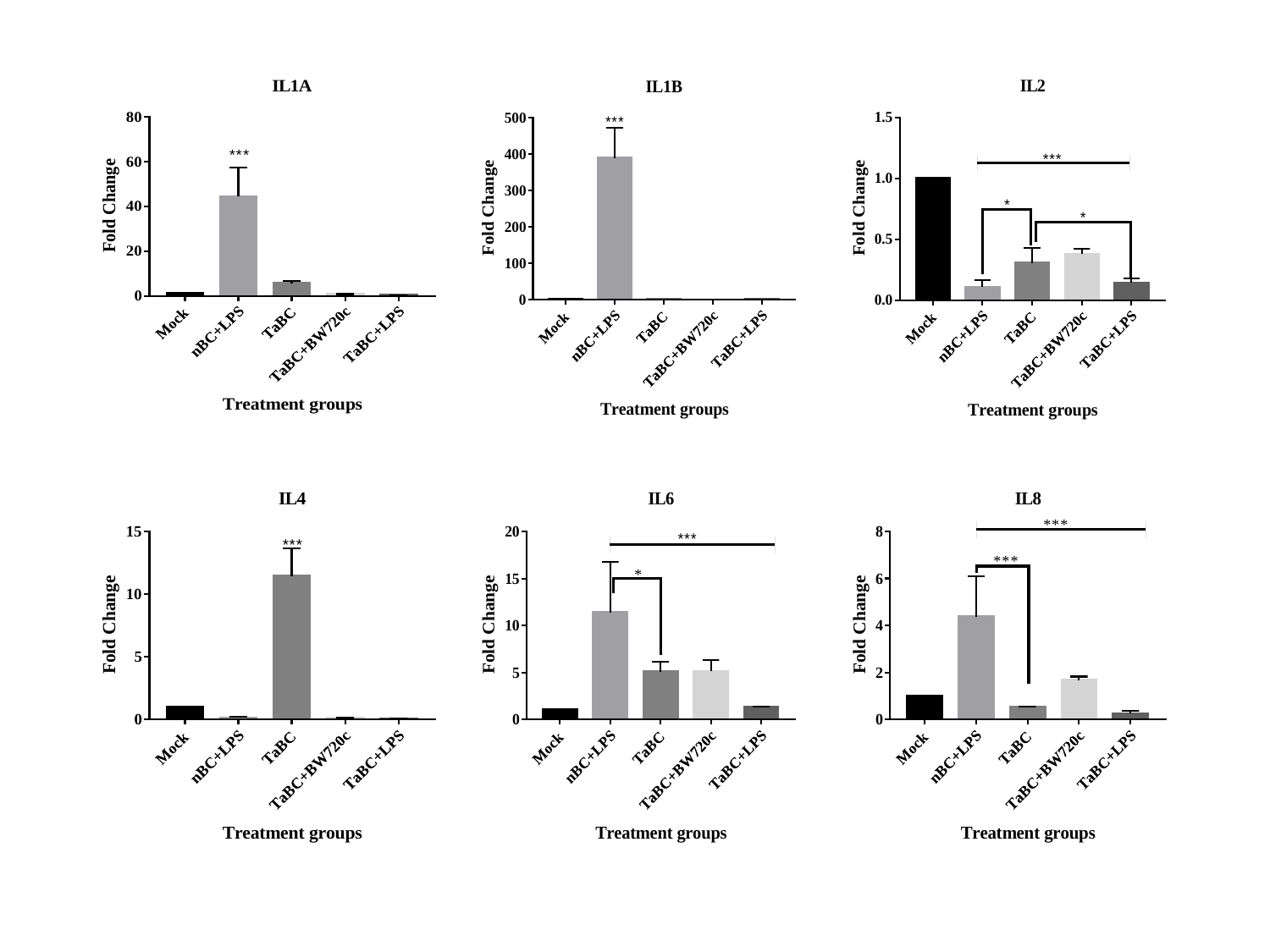

## Slide 2
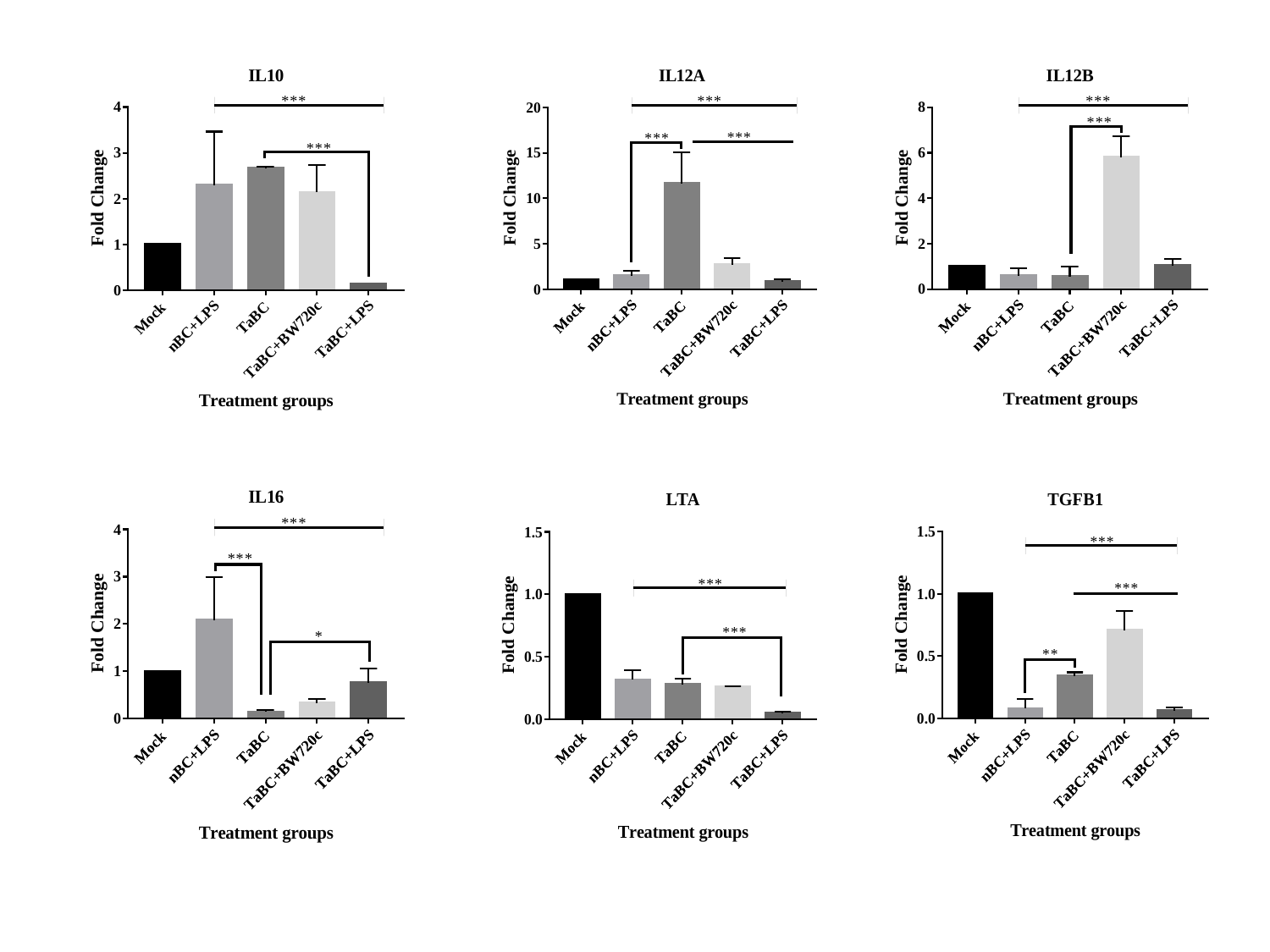

## Slide 3
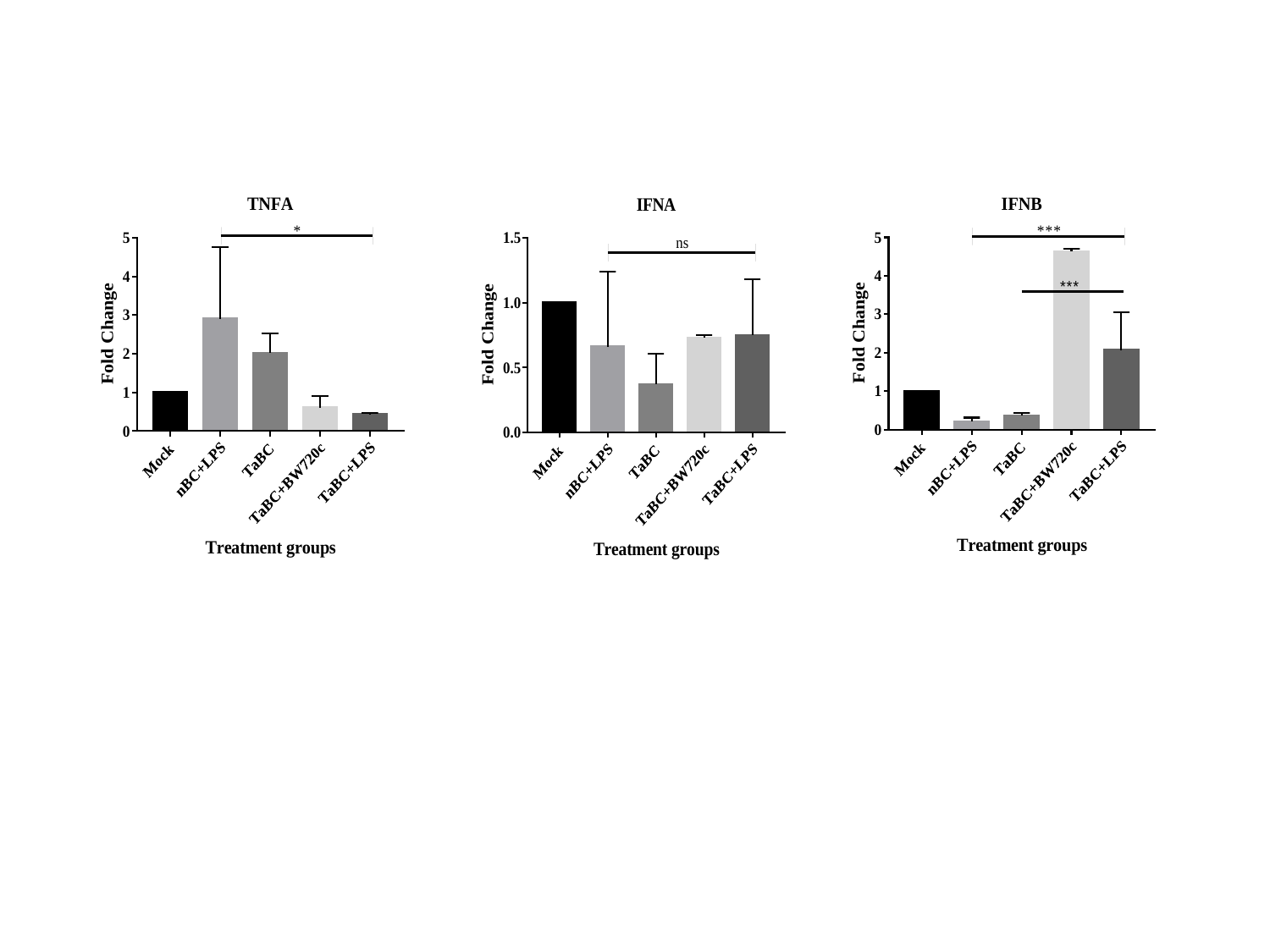

Supplement: Supplementary file 1 [file genes-10-00329-s001.pptx]
